# *Helicobacter pylori*: Genomic Insight into the Host-Pathogen Interaction

**DOI:** 10.1155/2015/386905

**Published:** 2015-02-05

**Authors:** Kathryn P. Haley, Jennifer A. Gaddy

**Affiliations:** ^1^Department of Medicine, Vanderbilt University School of Medicine, Nashville, TN 37232, USA; ^2^Tennessee Valley Healthcare Systems, US Department of Veterans Affairs, F523-ACRE Building, 1310 24th Avenue South, Nashville, TN 37212, USA

## Abstract

The advent of genomic analyses has revolutionized the study of human health. Infectious disease research in particular has experienced an explosion of bacterial genomic, transcriptomic, and proteomic data complementing the phenotypic methods employed in traditional bacteriology. Together, these techniques have revealed novel virulence determinants in numerous pathogens and have provided information for potential chemotherapeutics. The bacterial pathogen, *Helicobacter pylori*, has been recognized as a class 1 carcinogen and contributes to chronic inflammation within the gastric niche. Genomic analyses have uncovered remarkable coevolution between the human host and *H. pylori*. Perturbation of this coevolution results in dysregulation of the host-pathogen interaction, leading to oncogenic effects. This review discusses the relationship of *H. pylori* with the human host and environment and the contribution of each of these factors to disease progression, with an emphasis on features that have been illuminated by genomic tools.

## 1. Introduction

*Helicobacter pylori* is a Gram negative, spiral-shaped epsilonproteobacterium that colonizes half of the world's human population [1, 2].* H. pylori* is the dominant microorganism within the gastric niche and chronic infection with this pathogen is associated with increased risk for numerous negative disease outcomes including gastritis, peptic and duodenal ulcer, dysplasia, neoplasia, gastric B-cell lymphoma of mucosal-associated lymphoid tissue (MALT lymphoma), and invasive gastric adenocarcinoma [3].* H. pylori* persists in the gastric niche despite a robust immune response to infection, indicating that this pathogen has evolved elaborate mechanisms to evade both innate and adaptive arms of the human immune system [4].

**Figure 1:**
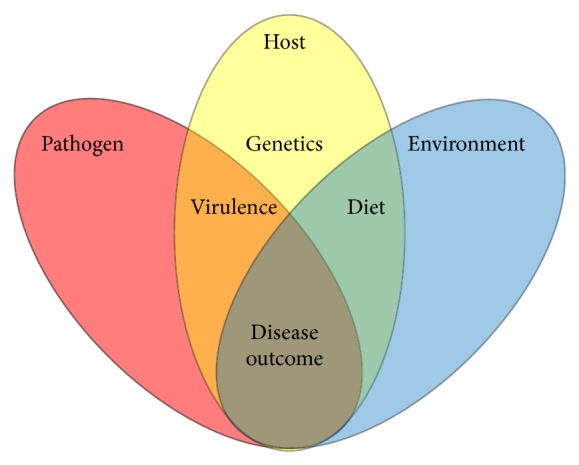
Model of factors influencing* H. pylori*-related disease outcome. Host genetics, environmental factors, and bacterial strain differences in virulence properties can all contribute to disease progression and increased risk of negative outcomes.

*H. pylori* typically colonizes the human stomach for years or even decades, often without adverse consequences [5]. Recent evidence indicates that there are health benefits associated with* H. pylori* colonization including protection from allergic airway disease, gastroesophageal reflux disease, Barrett's esophagus, esophageal adenocarcinoma, diarrheal disease, and obesity, implying that the relationship between* H. pylori* and its human host is complex and dynamic [6–8]. Conversely, numerous factors have been identified that can contribute to the development of negative outcomes with respect to* H. pylori* infection [9]. Together, these can be clustered into a triad of risk factors including host, pathogen, and environmental features that interact to promote disease progression (Figure 1). In recent years, epidemiologic studies, paired with genomic analyses, have shed light on specific interactions that are associated with increased risk of disease outcomes.

## 2. Evolution of* H. pylori* and Geographic Distribution of Strains

*H. pylori* is an ancient organism that has been prevalent within human populations for over 60,000 years [10]. Certain geographic areas, such as the Latin American Andes Mountain region, have high* H. pylori* infection rates and very high gastric cancer incidence, characteristics that coincide with low socioeconomic standards [11]. Interestingly, in other regions of the globe with similar socioeconomic conditions, including Africa, India, Thailand, Bangladesh, Pakistan, Iran, Israel, Malaysia, and Saudi Arabia, infection rates are high, but gastric cancer incidence is relatively low [12–14]. These are collectively referred to as “enigmas” within the published literature because the molecular mechanisms behind these differences remain largely obscure.

Genomics tools including whole genome sequencing, restriction fragment length polymorphism (RFLP) genome mapping, and analytical methods, such as maximum likelihood analysis and multilocus sequence typing (MLST), are enhancing the molecular epidemiological methods currently used to study* H. pylori *pathogenesis [15]. There is an impressive amount of genetic diversity between clinical isolates of* H. pylori* which is driven by a high mutation rate, frequent recombination events, and random genetic drift as well as positive Darwinian selection and fixation of base substitutions [16]. As human populations migrated across the globe their endemic* H. pylori *strains diverged alongside them leading to phylogeographic differentiation of this pathogen within human populations that can be classified into European, Amerindian, Asian, and African subgroups [17]. Frequently the phylogeographic origin of an* H. pylori *strain dictates specific host-adaptive responses through alterations in virulence factor expression. For example, European strains of* H. pylori* are frequently reported to have elevated virulence when compared to African strains, a characteristic that could explain the “African Enigma” [18]. A better understanding of the phylogenetic relationships between* H. pylori* strains could reveal novel mechanisms of virulence. Specifically, variations have been analyzed by MLST of housekeeping genes (*atpA*,* efp*,* ppa*,* mutY*,* ureI*,* trpC*, and* yphC*) to illuminate the genetic origins of* H. pylori* strains. These techniques have yielded results that have mapped the migration of humans in antiquity out of Africa, across Europe, through Asia, and into the Americas [19]. These analyses also suggest that* H. pylori* and human coevolution have been perturbed in some geographic areas. For example,* H. pylori* in India shares common ancestry with European* H. pylori* strains, indicating a possible acquisition of these strains during colonization by European imperial forces [20]. Conversely, MLST analyses of genomes of* H. pylori* from native Peruvians suggest that Amerindian strains of* H. pylori* persisted in these populations in the face of competition from Spanish* H. pylori* strains. It is likely that the Amerindian strains endemic to native Peruvians acquired Western isotypes of the* cag*-pathogenicity island, a European-derived virulence factor, resulting in a competitive advantage conferred to the Peruvian strains [21].

MLST analysis of housekeeping genes can provide insight into phylogeographic differentiation of these loci. Complementary to these techniques, investigations into virulence factors have shown that by evaluating both synonymous and nonsynonymous nucleotide substitutions within the coding region, positive selection for amino acid diversity can be determined. These changes in amino acid sequence can be associated with increased risk for peptic ulcer disease and can also be correlated with variations in geographic origin (Western or Asian) [22].

Besides undergoing phylogeographic differentiation, genomic analyses have revealed that* H. pylori *has, like other obligate human pathogens including* Chlamydia trachomatis* and* Mycoplasma pneumoniae*, undergone reductive evolution by reducing both the number of open reading frames (coding region sequences) and the total size of its genome [23–25]. This likely occurred as a consequence of its coevolution within the human host which provides a specialized niche for bacterial colonization and proliferation and consequently reduces the need to maintain genes involved in vital processes such as macronutrient synthesis and acquisition [26]. These findings underscore the importance of utilizing genomic tools to determine the “core genome” within* H. pylori *to better understand the basic metabolic requirements for prokaryotic life.

## 3. *H. pylori* Genomic Flexibility and Genetic Regulation

*H. pylori* exhibits unusual genetic flexibility and it is hypothesized that the variability within the genome could potentially account for the organism's ability to adapt to the dynamic environment within the host gastric niche, facilitating chronic colonization. These adaptations include both reversible and irreversible changes to the genome as well as regulatory mechanisms that modulate gene expression [27]. Analyses of numerous whole genome sequences indicate that* H. pylori* has evolved clusters of genes within genomic islands that harbor distinct areas of variability, termed “plasticity zones.” These plasticity zones are likely involved in horizontal gene transfer, a discovery that is supported by the presence of short conserved integration motifs and coding regions that are orthologous to integrating conjugative elements (ICEs). It is postulated that these ICEs, which are prevalent and widely distributed among all sequenced* H. pylori* strains, provide a fitness advantage to the bacterium by aiding genetic recombination events, which could ultimately promote immune evasion and increased ability to colonize, as well as other currently unappreciated alterations which would provide a selective advantage for the organism [27]. This hypothesis is supported by recent research which indicates that genetic modifications occur at a rate that is 10 times faster during acute or early infection when* H. pylori* is initially encountering the host's immune response than during chronic infection. These results also indicate that* H. pylori* mutation rates are far higher than any other bacteria currently assessed and that many of the mutations occur in genes that encode putative outer membrane proteins [28]. These proteins are implicated in host-pathogen interaction and transmission between hosts and are also likely targets for the adaptive immune response [29]. Thus, modification of these key surface-exposed antigens would likely alter these interactions to promote the establishment of chronic* H. pylori* infection and therefore the genes encoding these proteins experience the greatest selective pressure.

In addition to mapping genome sequence and structure, next-generation sequencing technology can now profile genome function by determining how and when genes are expressed and the regulatory networks that govern these expression subunits. Global transcriptomic analysis has revealed complexity in the riboregulation of* H. pylori *gene expression.* H. pylori* employs approximately 60 small RNAs as well as a surfeit of transcriptional start sites within operons, indicating uncoupling of polycistronic transcriptional regulation. Interestingly, about 5% of the open reading frames encode leaderless messages that lack the canonical translational initiation signals such as the Shine-Dalgarno sequence [30]. Comparison of these features to other Epsilonproteobacteria, such as* Campylobacter jejuni*, reveals a lack of conservation of operon organization and riboregulatory elements. Together, these results indicate that transcriptional rewiring occurs differently in* C. jejuni* and* H. pylori* to compensate for the genetic variations that occurred after these two species diverged from a common ancestor [31].

Epigenetics has emerged as an important area of study to better understand the subtle and complex nature of gene regulation. Epigenetic modifications such as DNA methylation carried out by DNA methyltransferases can have drastic effects on both genome architecture and gene expression.* H. pylori* encodes numerous DNA methyltransferases and single-molecule real-time sequence analyses of the methylome of closely related strains revealed great diversity in the methylation of target sequences. This result is attributed to variation in the specificity of the methyltransferase domain as well as variation within the methylation target sequence [32, 33]. Together, these features contribute to changes in gene regulation including modulating expression of* flgE* (encoding a flagellar component),* cagY* (encoding a type IV secretion component), and* ureC *(encoding a subunit of the urease complex) [32]. These results underscore the importance of expression dynamics and the necessity to identify the numerous regulators responsible for mediating complex interactions between the host and pathogen to further our understanding of chronic infectious processes.

## 4. Toxins Encoded in the* H. pylori* Genome

**Figure 2:**
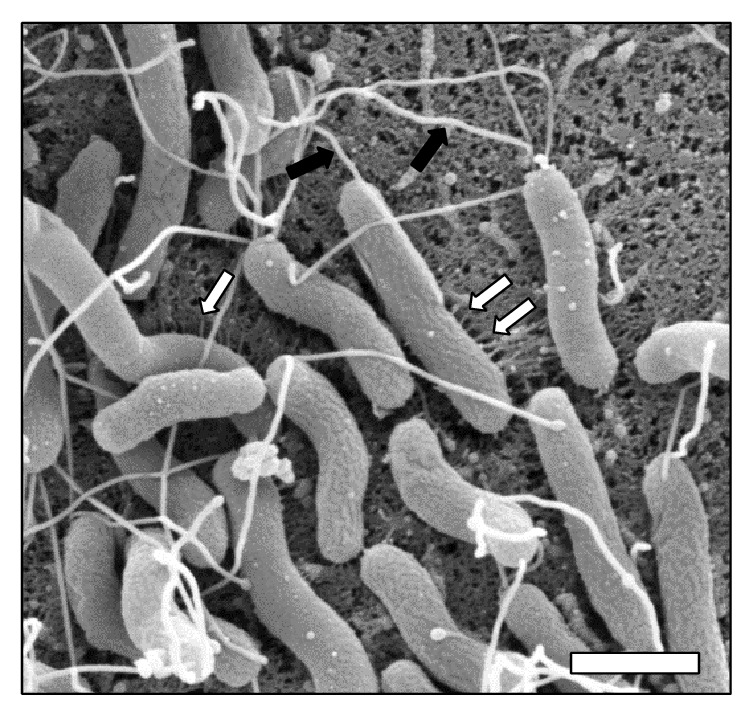
High resolution scanning electron micrograph of* H. pylori* in contact with human gastric epithelial cells.* H. pylori* virulence factors including flagella (black arrows) and* cag*-T4SS pili (white arrows) are present on the bacterial cell surface during host-pathogen interaction. Flagella aid in cell motility through the mucus layer to penetrate host tissues. The* cag*-T4SS pili induce proinflammatory and oncogenic cellular responses. Magnification bar indicates 1 *μ*m.

*H. pylori* exerts an immunomodulatory effect within this host tissue as a strategy to circumnavigate both innate and adaptive immune systems. Two toxins, the vacuolating cytotoxin (VacA) and the cancer-associated gene toxin (CagA), have been implicated in perturbing host immunological responses [34–36]. VacA is a pore-forming toxin secreted by* H. pylori* that causes a wide variety of alterations in host cell biology including cell vacuolation, autophagy, inhibition of T-cell proliferation, and induction of programmed necrosis [36–38]. The gene that encodes VacA has been shown to have variation between strains and the s1m1 variant is associated with the greatest risk for development of diseases including precancerous lesions and intestinal metaplasia [39, 40]. Sequencing data paired with epidemiological studies have revealed that polymorphisms within the intermediate region (i1-type) of VacA are associated with increased risk of peptic ulcer disease [40]. Additionally, evaluation of the molecular evolution of VacA reveals that positive selection has modified the sequence encoding VacA in a process that is independent of the evolution of the core genome [41]. This type of separate positive selection is also observed in the gene encoding the major surface antigen, CagA [41]. CagA is a cytotoxin that is translocated into host cells by the* cag*-type IV secretion system (*cag*-T4SS), a macromolecular nanomachine encoded by several genes within the* cag *pathogenicity island (*cag*-PAI) [42]. This T4SS is assembled at the host-pathogen interface (Figure 2) and is implicated in secretion of peptidoglycan and the aforementioned cytotoxin, CagA which results in numerous changes to host cell biology including upregulation of proinflammatory cytokines, alteration of actin cytoskeleton, disruption of metal homeostasis, and aberrant cell signaling [42–46]. Evaluation of* cagA* sequences has revealed that amino acid polymorphisms within the Glu-Pro-Ile-Tyr-Ala (EPIYA) segments contribute significantly to carcinogenesis [47]. This is interesting, because these regions are phosphorylated by host tyrosine kinases and are involved in the modulation of host signal transduction events. Together, these studies have revealed that polymorphisms within the coding regions for the cytotoxins VacA and CagA, specifically the i1 intermediate region and the EPIYA motif, respectively, can contribute to increased risk for development of gastric diseases [48, 49].

## 5. Expression of* H. pylori* Virulence Factors

Successful chronic infection of a vertebrate host is a delicate balance between host and pathogen. The pathogen expresses virulence factors to (1) elicit an immune response which eliminates resident microbiota, (2) acquire nutrients, (3) permit bacteria to penetrate host tissues, and (4) allow bacteria to turn the host tissue into a replicative niche. Bacteria have evolved to tightly control the expression of virulence factors to promote successful colonization.* H. pylori* has evolved to respond to environmental stimuli, such as gastric acid, with a repertoire of regulatory elements such as two-component systems comprised of a sensor kinase (ArsS) and a response regulator (ArsR), which ultimately modulates the expression of virulence genes involved in motility and* cag*-T4SS function [50]. Motility of* H. pylori* cells through the gastric mucosa is accomplished by utilization of numerous lophotrichous flagella (Figure 2). Once* H. pylori* penetrates the gastric mucosa, it interacts with host epithelial cells and elaborates* cag*-T4SS pili (Figure 2). The* cag*-T4SS encoded within the* cag*-PAI is organized into multiple overlapping operons that are likely coregulated as well as divergently regulated by numerous types of stimuli found within the gastric niche [51]. Besides pH,* H. pylori* senses diverse environmental cues including iron, nickel, cobalt, and zinc and responds to these cues by altering virulence expression [52]. For example, in conditions of low iron availability,* H. pylori* increases* cag*-T4SS activity and pilus biogenesis as well as expression of numerous flagellar components [53]. Conversely, in conditions of low zinc availability,* H. pylori *represses* cag*-T4SS machinery [54]. These data indicate that environmental cues present in the host can alter the carcinogenic potential of* H. pylori* and increase the risk of negative disease outcomes.

## 6. Dietary Contribution

Bacteria respond to their environment and alter virulence factor expression accordingly as described above. One of the numerous environmental stimuli that* H. pylori* encounters in the gastric niche are molecules derived from the host diet. Numerous dietary habits such as iron deficiency, salt preference, nitrite, protein, and fat intake have been epidemiologically linked with increased risk of* H. pylori*-related disease [55]. However, precious few of these dietary factors have been recapitulated in an animal model of* H. pylori* infection with some exceptions. Dietary iron deficiency has been correlated with increased risk of* H. pylori*-related disease progression [56]. In a rodent model of* H. pylori* infection, animals fed a low-iron diet exhibited higher incidences of gastric cancer compared to animals fed an iron-replete diet. Proteomics analyses of strains of* H. pylori* derived from these animals revealed that low-iron conditions induced expression of numerous virulence factors including flagellar proteins, a VacA paralog, CagA, HopQ, and urease [53]. Concomitantly, conditions of low iron availability increased* H. pylori *CagA T4SS induction of host proinflammatory IL-8 secretion, a result that correlated with the increase in gastric inflammation in animals fed a low-iron diet. Similarly, dietary salt intake has been associated with increased risk of gastric disease. In a rodent model of* H. pylori* infection, animals fed a high-salt diet exhibited higher incidences of gastric cancer and inflammation compared to animals fed a regular salt diet. Analysis of bacterial and host transcripts revealed that* cagA* and IL-1*β*, respectively, were highly upregulated in* H. pylori*-infected animals in response to dietary salt intake, a result that correlated with disease phenotypes [57]. Thus, variations in dietary ion consumption could lead to changes in bacterial virulence factor expression that ultimately alter disease progression.

## 7. Host Factors Associated with Disease

In addition to the numerous bacterial factors that have been demonstrated to affect disease outcome, genomic approaches have revealed host factors that are correlated with* H. pylori*-associated disease manifestation. MALT lymphoma has been characterized by microarray analyses which revealed pronounced infiltration of gastric tissue with CD4(+) T cells expressing CD28 and CD69 as well as an increased expression of calprotectin [58]. These results indicate that MALT lymphoma tumor cell proliferation is driven by Th2-polarized activated T cells and innate immune cells. Complementing this study, numerous subsequent studies have implicated Th1, Th17, and the host neutrophil-associated protein calprotectin as host molecules that are induced in response to* H. pylori* infection and associated with gastric inflammation [59]. Interestingly, whole genome expression profiles and sequencing revealed that polymorphisms in IL-1*β* and the IL-8 promoter region can increase the risk of* H. pylori*-related diseases such as gastric cancer [62, 63]. Both IL-1*β* and IL-8 are powerful proinflammatory cytokines implicated in gastric inflammation and carcinogenesis in response to* H. pylori*. There are numerous reports supporting that the dysregulation of the host-pathogen interaction, initiated through promotion of inflammation, demolishes resident microbiota to tip the balance in favor of a pathogen, ultimately resulting in disease progression [64, 65]. Aligned with these, genomic analyses have revealed that* H. pylori* has coevolved with its human host to promote less severe gastric lesions, and that disruption of the coevolution (introduction of nonnative strains of* H. pylori *into a human host) contributes to differences in disease severity [66, 67]. Together, these host-specific factors should be taken into account when crafting models of infectious disease risk.

## 8. Antibiotic Resistance Mechanisms

Once chronic infection is established, clearance of the colonizing bacteria requires administration of antimicrobial chemotherapeutics. The standard treatment for* H. pylori* infection is triple therapy with a proton pump inhibitor (PPI), amoxicillin, and clarithromycin, but current treatment paradigms in populations predominantly colonized with resistant strains favor quadruple therapy with the addition of metronidazole [68]. Antimicrobial resistance is a widespread problem among bacterial pathogens and recent evidence indicates the emergence of antibiotic-resistant strains of* H. pylori* in clinical samples. In an effort to better understand the molecular mechanisms that govern antimicrobial resistance, whole genome sequencing has been performed to characterize patterns of genotypic changes that can be correlated with phenotypic changes in antibiotic susceptibility [69]. The results indicate that resistance to clarithromycin can be conferred in* H. pylori* via mutations in 23S rRNA genes as well as modifications within genes that encode the TolC-family of efflux pumps [70]. Additionally, resistance to amoxicillin was conferred through mutations within outer membrane protein coding regions (*hopC*,* hofH*, and* hefC*) and penicillin-binding proteins (*pbp1* and* pbp2*) [71]. Metronidazole resistance has also emerged through frameshift and nonsense mutations in the* rdxA* or* frxA* coding regions or alternately in the ferric uptake regulator (*fur*) locus, rendering quadruple therapy ineffective in many cases [72]. In these instances where canonical triple or quadruple therapies fail, second line drugs such as tetracycline and fluoroquinolones are often employed. Unfortunately, both tetracycline and fluoroquinolone resistant isolates of* H. pylori* have emerged. Sequencing reveals that* 16S rRNA* substitutions confer tetracycline resistance, while* gyrA* mutation at codon 87 or 91 results in fluoroquinolone resistance [72, 73]. Collectively, these data demonstrate that* H. pylori* has the capacity to modify its genome to circumnavigate the selective pressure of antimicrobial chemotherapeutics. Understanding the genetic elements present in antibiotic-resistant strains of* H. pylori* could provide novel targets for new antimicrobial strategies that will restore the utility of existing therapies.

## 9. Conclusions

**Figure 3:**
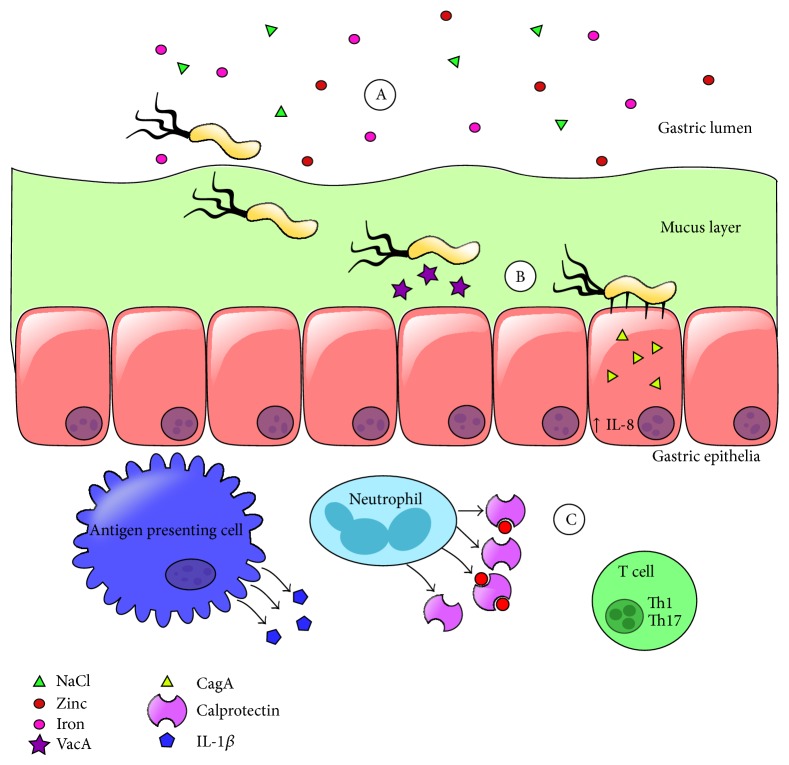
Model of* H. pylori*-host interaction* in vivo*. (A)* H. pylori* encounters numerous ions in the gastric niche and utilizes flagella to penetrate the mucus layer and reach the gastric epithelia. (B)* H. pylori* secretes VacA and CagA cytotoxins, causing changes in host cell biology. (C) The adaptive immune response as a consequence of* H. pylori* infection is skewed to expand Th1 and Th17 populations.

*H. pylori* is an ancient pathogen that has evolved to persistently colonize the human gastric niche. Infection with this pathogen leads to diverse outcomes ranging from asymptomatic gastritis to invasive adenocarcinoma. Factors that modulate risk fall into three major categories including pathogen attributes, host genetics, and environmental stimuli. Genomic tools have revealed numerous aspects of the host-pathogen interaction, including dietary ion consumption, expression of virulence factors such as toxins, secretion systems, and flagella, induction of proinflammatory signaling, and cytokine secretion (IL-1 and IL-8) which ultimately leads to adaptive immune responses including Th1 and Th17 expansion (Figure 3). These events at the host-pathogen interface could be exploited to influence the outcome of* H. pylori*-related diseases. Future work is required to develop chemotherapeutic strategies tailored to manipulate these interactions and tip the balance in favor of the health of the human host.
